# Optimization of Sample Processing for Droplet Digital PCR Quantification of *Campylobacter coli* and *Campylobacter jejuni* in Chicken Liver

**DOI:** 10.3390/pathogens15060638

**Published:** 2026-06-16

**Authors:** Joseph Capobianco, Chin-Yi Chen, Yiping He

**Affiliations:** Characterization and Interventions for Foodborne Pathogens Research Unit, Eastern Regional Research Center, Agricultural Research Service, United States Department of Agriculture, 600 East Mermaid Lane, Wyndmoor, PA 19038, USA; joe@lytostech.com (J.C.); chin-yi.chen@usda.gov (C.-Y.C.)

**Keywords:** *Campylobacter*, ddPCR, detection, quantification, chicken liver, foodborne pathogen, food safety, sample preparation

## Abstract

Accurate detection of *Campylobacter* in chicken liver is hindered by strong matrix inhibition. This study evaluated sample-processing strategies to improve droplet digital PCR (ddPCR) quantification of *Campylobacter coli* and *Campylobacter jejuni* in chicken liver. Mechanical homogenization (Stomacher) and enzymatic/mechanical dissociation (gentleMACS), with and without 8 μm filtration, were compared. Particle-size analysis showed that filtration, especially following gentleMACS treatment, produced smaller, more uniform particles and reduced variability. Percent-degradation assays confirmed that gentleMACS achieved substantially greater tissue disruption than Stomacher homogenization. The multiplex ddPCR assay, which simultaneously targets *C. coli* and *C. jejuni*, produced droplet counts comparable to single-target reactions, indicating minimal interference between targets under the conditions tested. In inoculated liver samples, gentleMACS processing yielded droplet counts similar to those obtained from pure cultures, whereas unprocessed liver caused severe matrix interference and inconsistent quantification. Furthermore, gentleMACS-treated samples exhibited strong log-to-log linearity for quantifying *C. coli* and *C. jejuni*, enabling detection near 1 genome copy equivalent per reaction. Overall, the results indicate that enzymatic/mechanical dissociation combined with fine-pore filtration improves ddPCR detection of *Campylobacter* species in chicken liver.

## 1. Introduction

*Campylobacter* spp., particularly *Campylobacter jejuni* and *Campylobacter coli*, are among the most common bacterial causes of foodborne gastroenteritis worldwide. In the United States alone, an estimated 1.3 million illnesses occur annually, making *Campylobacter* a major public-health concern [1,2,3,4]. Infection typically manifests as diarrhea, abdominal pain, fever, nausea, and vomiting, and while most cases are self-limiting, severe infections can require hospitalization. A small but significant proportion of cases progress to post-infectious sequelae such as Guillain–Barré syndrome, an autoimmune neuropathy that can result in long-term paralysis [5]. The substantial disease burden, combined with the potential for severe outcomes, underscores the need for accurate detection and quantification of *Campylobacter* in foods [6].

Poultry products are recognized as the primary reservoir for *Campylobacter*, with contamination occurring both on carcass surfaces and within internal organs [7]. Although the organism is most associated with the intestinal tract, multiple studies have demonstrated that poultry liver is frequently contaminated [8]. Surveys have reported contamination rates ranging from 40% to 78%, with concentrations reaching up to 1.8 log Colony Forming Unit (CFU)/g [9]. Because chicken liver is often incorporated into pâtés and other lightly cooked dishes, even low-level contamination can pose a significant risk to consumers [10]. The combination of high prevalence, variable contamination levels, and frequent undercooking makes poultry liver a particularly important matrix for *Campylobacter* surveillance.

In the United States, the Food Safety and Inspection Service (FSIS) of the U.S. Department of Agriculture (USDA) conducts routine monitoring of *Campylobacter* in raw poultry products. Under this program, samples collected from processing plants and retail establishments are analyzed using the USDA-FSIS Microbiological Laboratory Guidebook (MLG) 41.09 method [11]. This workflow includes enrichment, screening with the 3M Molecular Detection System (MDS) based on loop-mediated isothermal amplification (LAMP), and confirmation by selective plating, microscopy, latex agglutination, and biochemical assays. While this approach is effective for determining prevalence, it does not provide quantitative information on contamination levels.

Quantification of foodborne pathogens offers several advantages beyond presence/absence testing. Quantitative data enables processors and regulators to assess contamination trends, evaluate the effectiveness of interventions, and implement targeted corrective actions. For example, increases in *Campylobacter* levels at specific processing steps, such as post-chill, can prompt focused mitigation strategies. Quantitative data also improve the accuracy of microbial risk assessments, support evidence-based regulatory decisions, and help prioritize inspection and enforcement activities. For industry, quantification facilitates continuous improvement by enabling the tracking of intervention performance over time and identifying deviations that may require corrective action [12,13].

Several analytical methods are available for quantifying microbial contamination. The most probable number (MPN) method is widely used and statistically robust, relying on serial dilutions, selective enrichment, and Poisson-based inference [14]. However, MPN is labor-intensive, time-consuming, and susceptible to stochastic variation, particularly when bacterial loads are low or samples are highly heterogeneous [15]. Molecular methods such as quantitative PCR (qPCR) offer faster turnaround times but require standard curves and are sensitive to matrix-associated inhibitors.

Droplet digital PCR (ddPCR) provides an alternative approach that enables absolute quantification without standard curves [16]. By partitioning reactions into tens of thousands of nanoliter droplets and performing endpoint PCR in each partition, ddPCR calculates target concentrations based on the ratio of positive to negative droplets [17,18]. This partitioning improves tolerance to inhibitors and enhances precision, making ddPCR an attractive option for quantifying pathogens in complex matrices [19,20]. ddPCR has been successfully applied to quantify *E. coli*, *Listeria monocytogenes*, *Salmonella*, and other foodborne pathogens in a variety of food [21,22]. However, its performance is highly dependent on the generation of stable, uniform droplets. Residual particulates, lipids, and proteins, common in many food matrices, can disrupt droplet formation, suppress amplification, and compromise quantification accuracy [23,24].

Chicken liver presents additional challenges compared with other poultry matrices because *Campylobacter* can occur internally, the tissue supports bacterial survival, and the product is often undercooked. Its delicate structure also increases the risk of cross-contamination. In addition, liver tissue is a particularly difficult matrix for ddPCR due to its dense structure, high lipid content, and abundant cellular debris [25], all of which can reduce droplet counts, increase variability, and introduce quantification errors. Therefore, effective sample pretreatment is critical to minimize matrix interference and ensure reliable ddPCR performance. Mechanical homogenization methods such as Stomacher processing are widely used in food microbiology but may not sufficiently disrupt liver tissue to prevent interference in droplet-based assays [26].

The gentleMACS system offers a standardized platform that combines enzymatic and mechanical dissociation to generate uniform tissue suspensions. This technology has been successfully used to isolate viable hepatocytes from rodent liver and has demonstrated utility across diverse biological applications, including cell isolation, tissue digestion, and microbial recovery [27]. Prior studies suggest that enzymatic pretreatment of food matrices does not compromise detection assays and may enhance pathogen recovery, supporting the potential value of enzymatic/mechanical dissociation for ddPCR-based pathogen detection [28,29].

Although ddPCR offers advantages for pathogen quantification, its application to complex liver matrices is still limited by matrix-associated inhibition, poor droplet stability, and inadequate tissue disruption. To date, few studies have systematically evaluated whether tissue dissociation strategies, particularly enzymatic/mechanical approaches, can reduce matrix interference and improve ddPCR performance in poultry liver.

Therefore, the objective of this study was to compare an enzymatic/mechanical dissociation approach with conventional mechanical homogenization for enhancing ddPCR-based quantification of *C. coli* and *C. jejuni* in chicken liver. Specifically, the study aimed to determine whether gentleMACS-based dissociation can reduce matrix-associated inhibition and produce more accurate and consistent quantification relative to traditional methods.

## 2. Materials and Methods

### 2.1. Whole Liver Preparation

Chicken livers were obtained from multiple local retail stores, stored at 4 °C, and processed within 24 h of purchase to maintain sample integrity and minimize microbial changes unrelated to the experimental treatments. A multiplex qPCR assay [30] was used to verify that all samples were free of endogenous *Campylobacter* contamination. The livers were then aseptically sectioned into 1.2 g portions following a previously described method [28] to ensure that each subsample was representative of the whole organ while providing sufficient tissue for downstream processing, inoculation, and ddPCR analysis.

### 2.2. Liver Rinse Preparation

**Figure 1:**
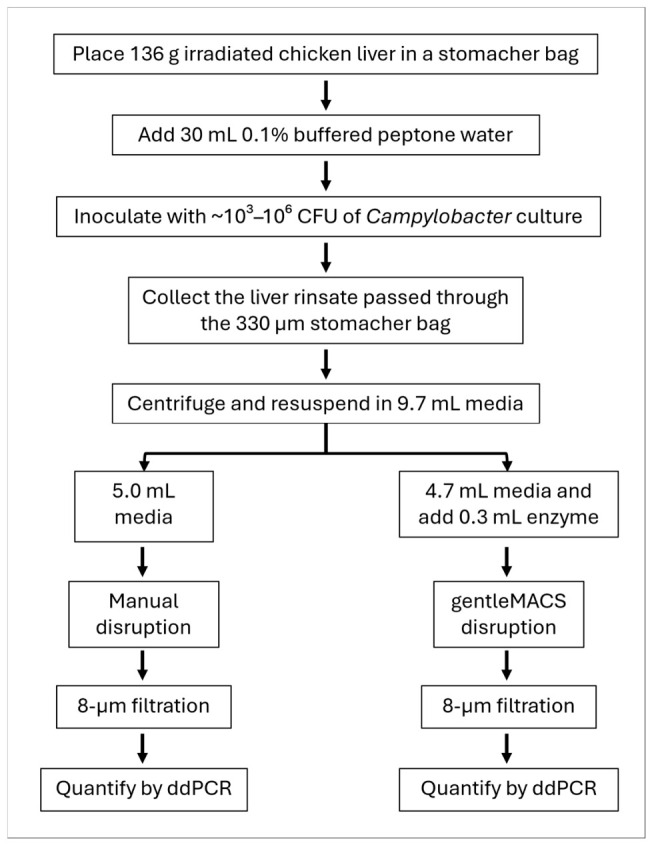
Flow chart illustrates the sample-processing and analytical workflow for quantitative detection of *Campylobacter* in chicken liver. For each treatment, liver samples were prepared and analyzed in triplicate.

Liver rinse was prepared based on FSIS Directive 10250.1 [31], with the modifications described below. Irradiated chicken liver was used to ensure the absence of viable endogenous *Campylobacter* or other background microbiota that interfere with accurate quantification of inoculated CFU during experimental treatments. The chicken livers were irradiated at the National Center for Electron Beam Research, Texas A&M University System, using approximately 25 kGy E-beam treatment to inactivate microorganisms present in the tissues. Portions of the irradiated chicken liver (136 g) were placed into a filtered stomacher bag with a 330 μm partition (Whirl–Pak, Madison, WI, USA), combined with 30 mL of 0.1% buffered peptone water (BPW). The resulting rinse was collected through the integrated filter, transferred to a 50 mL tube, and centrifuged at 10,000 RCF for 10 min at room temperature. After centrifugation, the BPW supernatant was decanted, and the pellet was immediately resuspended in Bolton Broth supplemented with 5% laked horse blood (BB-HB) for subsequent treatment and analyses (Figure 1).

### 2.3. GentleMACS Treatment

Enzymatic and mechanical dissociation of liver material was performed using a commercial liver dissociation kit with the gentleMACS™ Octo Dissociator with heaters (Miltenyi Biotec, Auburn, CA, USA). Whole liver samples (1.2 g), prepared in triplicates, were transferred to C-tubes containing 4.7 mL of Bolton Broth supplemented with 5% laked horse blood (BB-HB). For liver rinse samples, the entire pellet was dispersed in 4.7 mL of BB-HB and transferred to a C-tube supplemented with 0.32 mL of Miltenyi’s enzyme cocktail. This cocktail contained Enzyme D (200 μL), Enzyme R (100 μL), and Enzyme A (20 μL). The enzymes were prepared according to the manufacturer’s instructions by reconstituting one vial of lyophilized Enzyme D, R, and A in 3 mL, 2.7 mL, and 1 mL of sterile 0.1% BPW, respectively. The C-tubes were loaded into the gentleMACS heater sleeves, and sample dissociation was carried out using the program 37C_m_LIDK_1. The chicken rinse samples not processed with the gentleMACS system were manually mixed by pipetting.

### 2.4. Stomaching Treatment

For thermal treatment prior to dissociation, 1.2 g of chicken liver and 4.7 mL of BB-HB were added to each stomacher bag with mesh dividers (pore size 330 μm) and incubated at 37 °C for 45 min to account for the heating provided by the gentleMACS cycle. After incubation, samples were homogenized in the Seward Stomacher 3500 (Seward Laboratory Systems Inc., West Sussex, UK) for 30 s at 150 rpm to ensure uniform dispersion of tissue and broth, which were experimentally optimized in our laboratory for efficient microbial recovery. Triplicate liver samples were prepared for the treatment.

### 2.5. Particle Size Determination

Dynamic light scattering (DLS) was used to evaluate sample particle size using a Mastersizer 3000 equipped with dual light sources and small volume sample dispersion unit (SVDU) (Malvern Panalytical Ltd., Westborough, MA, USA) Constant optical and physical parameters for both samples and solvent were selected from established database or literature sources [32]. Nanopure water was prepared using a Barnstead ultrapure water purification system.

Before sample measurements, 130 mL of nanopure water was added to the SVDU and circulated to establish instrument baseline conditions. The laser system was aligned, and a background measurement was obtained. To achieve the recommended obscuration range, 200 μL of liver suspension was added to 130 mL of nanopure water. The suspension was recirculated and particle size measurements were collected. Ten measurements were obtained for each of the three biological replicates. Data were analyzed using the manufacturer’s software, which applies a general-purpose model based on Mie theory to estimate particle size distributions. The analysis generated a volume-based particle size distribution spanning approximately 10 nm to 3500 μm. The Mastersizer 3000 also provided particle concentration values on a per-volume basis.

### 2.6. Percent Degradation Assessment

Percent degradation, defined as the weight loss relative to the initial mass, was assessed using a procedure adapted from a previously described method [28]. Briefly, samples were processed in triplicate. Each sample was vacuum-filtered through 8 μm filter paper (Whatman, Maidstone, UK), and the retained material was dried in an oven at 80 °C until its mass stabilized. Untreated controls were included to account for weight loss due to dehydration rather than enzymatic digestion or mechanical degradation. Each biological replicate was weighed five times. For each control, the dried retentate mass was subtracted from the initial 1.2 g to determine dehydration-related loss. Percent degradation was calculated using the formula: [(Initial − Final)/Initial] × 100 [29]. All statistic data analyses were carried out using JMP v19.0.4 software.

### 2.7. Campylobacter Culture

*C. coli* YH509 (GenBank accession number: CP172390) and *C. jejuni* YH 009 (CP131444) [33], originally isolated from retail chicken meat or liver products, was recovered from frozen stocks and streaked onto Brucella agar for overnight growth. Fresh colonies were then transferred into Brucella broth to obtain actively growing cultures. Overnight broth cultures were enumerated on Brucella agar for comparison with ddPCR-based quantification. All cultures were incubated overnight at 42 °C under microaerophilic conditions generated using an EZ Campy Container System Sachet (Becton, Dickinson and Company, Franklin Lakes, NJ, USA) within an airtight container [29].

Liver rinse samples, prepared in triplicate, were inoculated with fresh *C. coli* and *C. jejuni* cultures (approximately 10^3^–10^6^ CFU per sample) as described above. The CFU of the inocula was determined using the 6 × 6 drop plate method [34]. Following artificial contamination, cell-containing liver rinses were processed by gentleMACS dissociation and 8 μm filtration prior to ddPCR analysis. Whole bacterial cells were used directly for ddPCR; no DNA extraction step was performed (Figure 1).

### 2.8. Droplet Digital PCR (ddPCR)

A total of 22 μL of PCR mixture containing the *hipO* and *cdtA* primer-probe sets, targeting *C. jejuni* and *C. coli*, respectively, was prepared as shown in Table 1. The inclusivity and exclusivity of the assays were previously validated against multiple *Campylobacter* species and common foodborne bacteria [30]. Primers specific to *C. jejuni* and *C. coli* were synthesized by IDT (Integrated DNA Technologies, Coralville, IA, USA). Fluorescent probes labeled with FAM or HEX were obtained from Biosearch Technologies (Novato, CA, USA).

Droplets were generated by combining 20 μL of sample with 70 μL of droplet generation oil using an automated droplet-making instrument QX200 (Bio-Rad, Hercules, CA, USA). PCR amplification of 40 μL of the resulting emulsified reaction was performed on a CFX96 Deep Well thermocycler (Bio-Rad), in triplicate. The thermocycling program consisted of an initial enzyme-activation phase (95 °C, 10 min), followed by 40 sequential cycles of denaturation (95 °C, 30 s) and annealing/extension (58 °C, 1 min), and concluded with a final enzyme-stabilization step (98 °C, 10 min and then 4 °C, 10 min) prior to droplet analysis. Following amplification, droplets were then read using the QX600 droplet reader (Bio-Rad). The droplet acceptance thresholds, fluorescence-thresholding criteria, rain handling, and partition-classification strategy followed the manufacturer’s default settings in QuantaSoft v2.0 (Bio-Rad). No-template controls (NTCs) consistently produced zero positive droplets, confirming the absence of contamination and supporting the suitability of the default parameters. Because some samples contained inhibitors that reduced droplet counts below the software’s minimum threshold, genome copy numbers were calculated manually in Excel using the Poisson equation:

Genome copies per reaction = [−ln(1 − P/T)]/Vd×22

where *P* is the number of positive droplets, *T* is the total number of droplets generated, *Vd* is the individual droplet volume (μL), and 22 is the total reaction volume (μL). Manual values matched software-generated results from acceptable wells, indicating no systematic bias. Linear regression analysis was performed using JMP.

## 3. Results

### 3.1. Particle Size Distribution of Whole Liver and Liver Rinse

To improve *Campylobacter* detection in chicken liver samples, we evaluated several sample-processing methods prior to ddPCR analysis. Specifically, we compared mechanical homogenization using a Stomacher with enzymatic/mechanical dissociation using the gentleMACS Dissociator for whole liver samples, as well as manual dispersion with gentleMACS dissociation for liver rinse samples. Independent tests were performed on both whole liver and liver rinse samples, each comprising three biological replicates. A schematic overview of the experimental workflow is provided in Figure 1.

**Figure 2:**
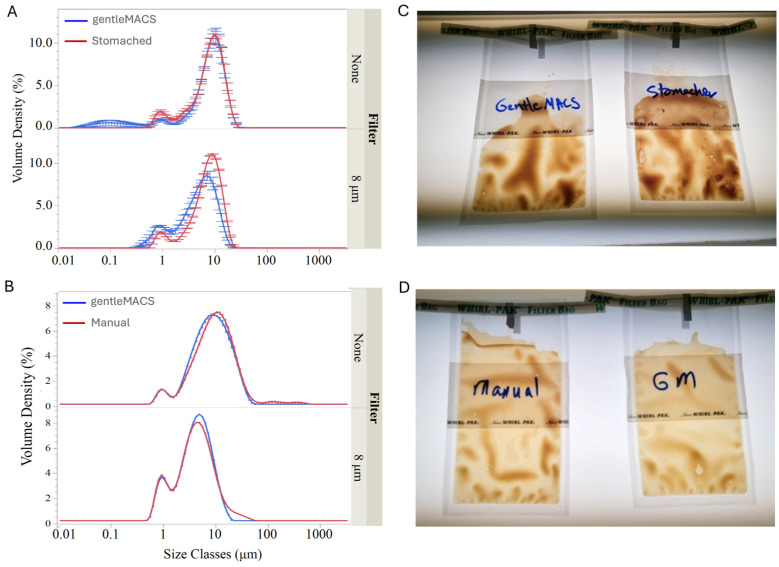
Particle size distribution of whole-liver (**A**) and liver-rinse (**B**) preparations. The plots show volume density (%) on the y-axis versus particle size class (μm) on the x-axis. Each curve represents the mean of 30 measurements obtained from 10 measurement replicates for each of three biological replicates; error bars indicate the standard deviation of the mean. In panels (**A**,**B**), the top graphs display particle-size distributions measured without filtration. The bottom graphs show distributions from material that passed through an 8 μm filter. Panel (**C**) presents the physical appearance of whole-liver samples after Stomacher homogenization and enzymatic/mechanical gentleMACS dissociation. Panel (**D**) shows liver-rinse samples following manual dispersion and enzymatic/mechanical gentleMACS™ dissociation.

To characterize the physical effects of each sample preparation method, we measured particle size distributions for whole liver samples processed by either Stomacher homogenization or gentleMACS dissociation, with and without an 8 μm filtration step (Figure 2A). Both Stomacher and gentleMACS treatments produced a broad and largely overlapping particle size profiles in unfiltered samples, indicating comparable levels of mechanical disruption. However, filtration of gentleMACS-treated samples shifted the distribution toward markedly smaller particles, demonstrating that enzymatic/mechanical dissociation generated finer fragments capable of passing through the 8 μm filter. No significant differences were observed between Stomacher- and gentleMACS-treated samples in the absence of filtration.

Particle-size analysis of liver-rinse preparations revealed similar trends (Figure 2B). Unfiltered rinses contained a wide range of particle sizes, including prominent peaks corresponding to large tissue fragments, and exhibited substantial variability among biological replicates. Filtration effectively removed these larger particulates, yielding narrower, more uniform distributions and reducing replicate-to-replicate variation. These results show that filtration substantially improves consistency in liver-rinse preparations, particularly when combined with gentleMACS dissociation.

Representative images of whole-liver and liver-rinse samples following Stomacher homogenization or gentleMACS dissociation are shown in Figure 2C,D. No visible differences were observed between the two treatments.

Collectively, these findings indicate that filtration is the key driver of uniformity, but gentleMACS enhances the effect by producing finer particles that pass through the filter more consistently.

### 3.2. Percent Degradation of Liver Tissue

**Figure 3:**
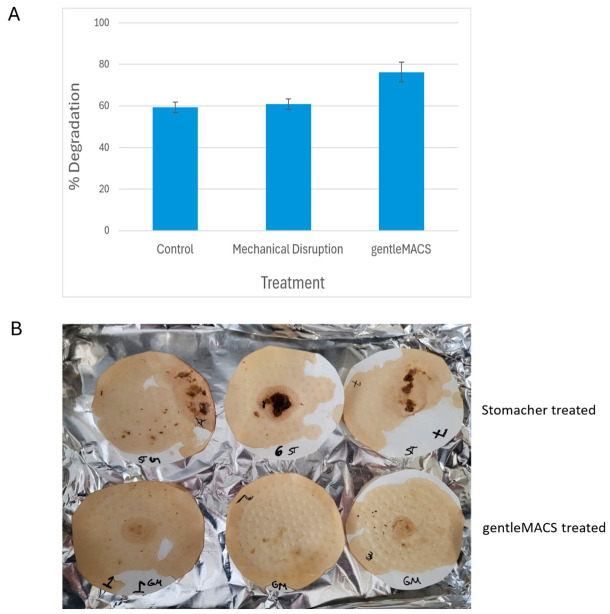
Degradation of chicken liver following Stomacher and gentleMACS processing. (**A**) Percent degradation (%) plotted against treatment type. Bars represent the mean of three biological replicates for Stomacher- and gentleMACS-treated samples; the untreated control includes six biological replicates to account for weight loss due to dehydration. Each biological replicate was weighed five times, and error bars indicate the standard deviation of the mean. The gentleMACS-treated samples are significantly different from the Stomacher-treated and untreated controls, with *p*-values < 0.0002 according to a Student’s *t*-test (α = 0.05). (**B**) Representative images of dried whole-liver samples (1.2 g) retained on 8 μm filters following gentleMACS or Stomacher processing.

To further evaluate the extent of tissue disruption achieved by each processing method, we next assessed potential degradation following treatment and drying (Figure 3A). GentleMACS-treated samples exhibited significantly greater degradation than both Stomacher-treated samples and untreated controls (Student’s *t*-test, *p* < 0.0002, α = 0.05), demonstrating that enzymatic/mechanical dissociation was the most effective approach for breaking down liver tissue. No significant difference was observed between untreated and Stomacher-treated samples.

Visual inspection of dried residues on 8 μm filters supported these findings (Figure 3B). GentleMACS-treated samples left noticeably less residual material than Stomacher-treated samples, consistent with more complete tissue degradation. These observations align with the smaller particle sizes observed in gentleMACS-processed samples (Figure 2A).

### 3.3. ddPCR Quantification of C. coli and C. jejuni Pure Cultures

**Figure 4:**
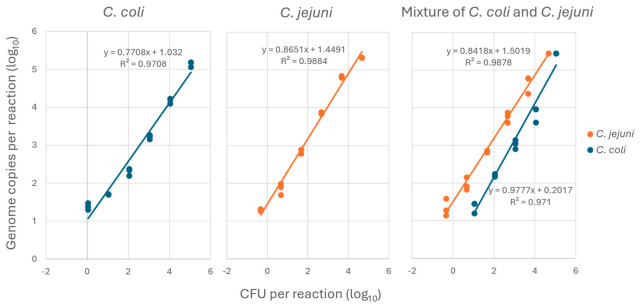
Multiplex ddPCR quantification of pure *C. coli* and *C. jejuni* cultures. Genome copy numbers measured by ddPCR are plotted on the y-axis against inoculated CFU on the x-axis for *C. coli*, *C. jejuni*, and mixed cultures. Reactions consistently produced ~20,000 droplets, and both targets were efficiently coamplified without evidence of competition. Linear regression analyses of the three biological replicates showed strong agreement between the measured and expected concentrations across ~10^0^–10^5^ cells per reaction, demonstrating robust sensitivity and precision in matrix-free conditions.

Having verified that liver tissue degradation was not a confounding variable, we proceeded to evaluate the baseline performance of the multiplex ddPCR assay using pure cultures. To characterize assay sensitivity and dynamic range, the ddPCR assay was evaluated using 10-fold serial dilutions of pure *C. coli*, *C. jejuni*, and mixed cultures, with each dilution tested in triplicate (Figure 4). Multiplex reactions consistently produced approximately 20,000 droplets, comparable to single-target assays, indicating that simultaneous amplification of both species neither impaired droplet formation nor introduced detectable competition.

In both pure and mixed cultures, the assay maintained linear quantification from ~10^0^ to 10^5^ cells per reaction and achieved detection limits of ~1 cell for each species. Because each *Campylobacter* cell contains a single copy of the target gene in its chromosome, the genome copy number measured by ddPCR corresponds directly to cell numbers. Log-to-log regression of measured genome copies against inoculated CFU yielded R^2^ values exceeding 0.97, demonstrating strong linearity and agreement between expected and measured concentrations. These results confirm that the multiplex ddPCR assay is sensitive, efficient, and robust in the absence of food matrices.

### 3.4. Multiplex ddPCR Detection of C. coli and C. jejuni in Artificially Contaminated Chicken Livers

After establishing the assay’s analytical performance in pure cultures, we sought to determine whether these results translated effectively to a more complex biological matrix. To extend ddPCR-based quantification of *Campylobacter* to food matrices, chicken livers were artificially contaminated with mixed cultures of *C. coli* and *C. jejuni* at varying concentrations. Following processing with or without gentleMACS dissociation, the resulting liver rinse samples were subjected to multiplex ddPCR analysis to assess assay performance under realistic matrix conditions.

**Figure 5:**
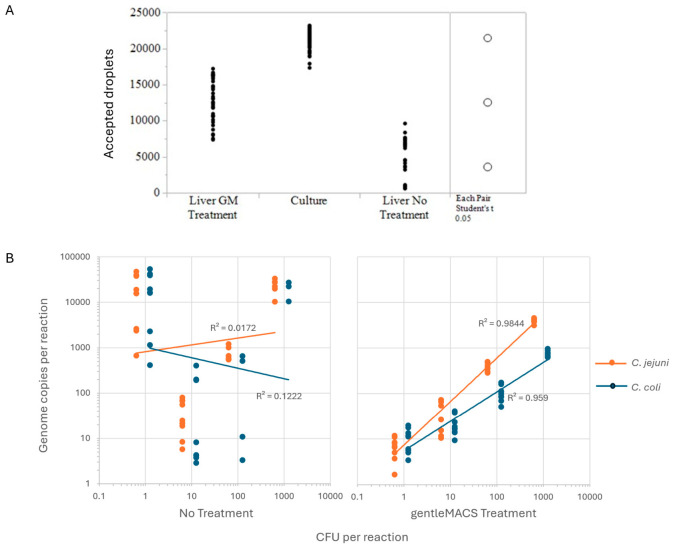
Multiplex ddPCR performance in artificially contaminated chicken liver following gentleMACS dissociation. (**A**) Number of accepted droplets generated from gentleMACS-treated and untreated liver samples compared with pure-culture reactions. GentleMACS-treated samples produced an average of 12,543 droplets, comparable to pure cultures (21,466 droplets), whereas untreated liver generated only 3593 droplets, indicating substantial matrix interference. (**B**) ddPCR-measured genome copies per reaction plotted against inoculated CFU for *C. coli* and *C. jejuni*. GentleMACS-treated samples exhibited strong log-to-log linearity (R^2^ = 0.96 for *C. coli*; R^2^ = 0.98 for *C. jejuni*) and detection sensitivity near 1 genome copy equivalent per reaction. Three biological replicates and three technical replicates were included in the assay.

One-way Anova showed that gentleMACS-treated liver samples generated a mean of 12,543 accepted droplets (95% confidence interval [CI], 12,008, 13,078), compared with only 3593 droplets from untreated samples (95% CI, 3051, 4135), indicating a substantial improvement in droplet formation (Figure 5A). However, droplet counts in treated samples remained lower than those observed in pure-culture reactions, which generated a mean of 21,466 droplets (95% CI, 20,955, 21,977). Overall, these results emphasize the importance of tissue dissociation in preparing food samples for ddPCR assay and show that gentleMACS treatment markedly enhances droplet formation and improves overall assay compatibility. Plating of gentleMACS-treated samples further confirmed that gentleMACS treatment did not reduce *Campylobacter* cell viability.

Figure 5B shows the quantification of *Campylobacter* genome copies per ddPCR reaction in artificially contaminated chicken livers, either treated or untreated with gentleMACS. The gentleMACS-treated samples exhibited strong linearity in log-to-log regressions of ddPCR-measured genome copies versus inoculated CFU, yielding R^2^ values of 0.96 for *C. coli* and 0.98 for *C. jejuni*. These results indicate good agreement between measured and input concentrations, with detection sensitivity of approximately 1 genome copy equivalent per reaction. In contrast, untreated liver samples showed poor droplet formation and inflated quantification estimates, reflecting substantial matrix-associated interference.

These results demonstrate that while ddPCR is inherently capable of detecting *Campylobacter* at concentrations near 1 genome copy equivalent per reaction, reliable detection in liver matrices requires effective sample preparation. GentleMACS dissociation markedly improves droplet formation and measurement accuracy, enabling robust quantification across the assay’s dynamic range.

## 4. Discussion

Effective detection of *Campylobacter* in chicken liver is hindered by the complex and inhibitory nature of liver tissue, which disrupts droplet formation and compromises the accuracy of ddPCR. In this study, we systematically evaluated multiple sample processing strategies to identify conditions that improve tissue disruption, reduce variability, and enhance compatibility with ddPCR-based quantification.

Liver tissue poses several inherent challenges for ddPCR. High viscosity, abundant cellular debris, and emulsified lipids interfere with the emulsification process required for stable droplet formation. Large or irregular particulates can rupture droplets during thermal cycling or impede their optical classification, while matrix-associated inhibitors reduce the number of accepted droplets and increase the likelihood of ambiguous partitions. These effects are consistent with inhibitory mechanisms reported in other complex matrices, such as high-fat foods and environmental samples, where ddPCR performance is often constrained by physical rather than purely chemical inhibition [20,23,35].

Our results demonstrate that enzymatic/mechanical dissociation using the gentleMACS system, particularly when combined with an 8 μm filtration step, substantially reduces matrix-related inhibition. GentleMACS processing decreases tissue viscosity and improves particle-size uniformity, whereas the 8 μm filter removes large residual particulates that interfere with emulsion formation. These improvements yielded higher accepted-droplet counts and more reliable quantification than untreated or mechanically homogenized liver rinses. Although filtration enhances clarity and droplet generation, it may also result in loss of target cells retained on the filter, potentially leading to underestimation of target levels. Because unfiltered samples did not produce enough droplets for direct recovery comparisons, this potential filtration-associated loss should be considered when interpreting ddPCR results from complex tissue matrices.

Baseline characterization of the multiplex ddPCR assay using pure cultures confirmed high analytical sensitivity, broad dynamic range, and no detectable target competition under matrix-free conditions. This benchmark allowed us to attribute performance declines in untreated liver samples to matrix interference rather than intrinsic assay limitations.

When applied to artificially contaminated liver, gentleMACS dissociation produced substantial gains in ddPCR compatibility. Treated samples generated far more accepted droplets than untreated samples, reflecting reduced viscosity and debris load. In untreated liver, low droplet numbers caused instability in the ratio of positive to negative partitions, yielding inflated concentration estimates, particularly at low inoculation levels, a known artifact when droplet counts fall below recommended thresholds. These findings underscore the importance of effective tissue dissociation for ddPCR accuracy in complex food matrices. Although gentleMACS treatment markedly improved droplet formation, treated liver samples still produced fewer droplets than pure-culture controls, likely due to residual particulates and matrix-associated physical effects that continue to interfere with emulsification. Importantly, mean droplet yields of approximately 12,500 remained well within the acceptable operational range for reliable Poisson-based quantification. The consistently narrow confidence intervals (95% CI, 12,008–13,078 droplets) indicate that tissue dissociation improved droplet generation without compromising quantification precision. Further optimization may help narrow this gap, but current droplet counts supported reliable quantification under the conditions evaluated.

Matrix-associated inhibition in this study was inferred primarily from reductions in accepted droplet counts and shifts in droplet amplitude distributions, both recognized indicators of partial inhibition in ddPCR workflows. However, these indirect measures do not quantify inhibition as precisely as an internal amplification control (IAC) or exogenous spike-recovery assay. Future validation studies will incorporate an IAC or spike-in control to more explicitly measure inhibition and better characterize matrix-dependent effects on assay performance.

In addition, the study relied on artificially contaminated samples and did not include naturally contaminated chicken liver, which may contain additional microbial or biochemical inhibitors. Only one isolate each of *C. coli* and *C. jejuni* was tested, limiting assessment of strain-to-strain variability. Furthermore, no interlaboratory assessment was conducted, nor was a direct comparison with conventional qPCR methods included. These limitations define important directions for future validation and standardization.

## 5. Conclusions

This study demonstrates that enzymatic/mechanical gentleMACS dissociation combined with fine-pore (8 μm) filtration improves ddPCR performance for quantifying *Campylobacter* spp. in chicken liver matrices. This workflow reduces matrix interference, stabilizes droplet generation, supports consistent quantification across the assay’s dynamic range, and enables accurate detection of *Campylobacter* at levels as low as ~1 genome copy equivalent per reaction in chicken liver. These findings indicate that gentleMACS-based processing can improve analytical performance in complex food matrices. However, additional validations across other food matrices, naturally contaminated samples, and interlaboratory settings are needed to fully establish scalability and generalizability.

## Figures and Tables

**Table 1:** Components and reaction volumes used in the ddPCR mixture.

Material	Primer/Probe Sequence	Final Concentration	Working Solution	Adding Volume
ddPCR supermix for probes		1×	2×	11 μL
*hipO-F*	5′-TCCAAAATCCTCACTTGCCATT	200 nM	10 μM	0.44 μL
*hipO-R*	5′-TGCACCAGTGACTATGAATAACGA	200 nM	10 μM	0.44 μL
*hipO*-FAM	5′-TGCAACCTCACTAGCAAAATCCACAGCT	200 nM	10 μM	0.44 μL
*cdtA*-F	5′-TGTCAAACAAAAAACACCAAGCTT	200 nM	10 μM	0.44 μL
*cdtA*-R	5′-CCTTTGACGGCATTATCTCCTT	200 nM	10 μM	0.44 μL
*cdtA*-Hex	5′-AAAATTTCCCGCCATACCACTTGTCCC	200 nM	10 μM	0.44 μL
Sample				2.2 μL
ddH_2_O				6.16 μL
Total volume				22 μL

## Data Availability

The original contributions presented in this study are included in the article. Further inquiries can be directed to the corresponding author.
